# Isolation of *Candidatus* Bartonella rousetti and Other Bat-associated Bartonellae from Bats and Their Flies in Zambia

**DOI:** 10.3390/pathogens9060469

**Published:** 2020-06-13

**Authors:** Yongjin Qiu, Masahiro Kajihara, Ryo Nakao, Evans Mulenga, Hayato Harima, Bernard Mudenda Hang’ombe, Yoshiki Eto, Katendi Changula, Daniel Mwizabi, Hirofumi Sawa, Hideaki Higashi, Aaron Mweene, Ayato Takada, Martin Simuunza, Chihiro Sugimoto

**Affiliations:** 1 Hokudai Center for Zoonosis Control in Zambia, Hokkaido University Research Center for Zoonosis Control, Sapporo 001-0020, Japan; yongjin_qiu@czc.hokudai.ac.jp (Y.Q.); kajihara@czc.hokudai.ac.jp (M.K.); harima@czc.hokudai.ac.jp (H.H.); hidea-hi@czc.hokudai.ac.jp (H.H.); 2 Division of Global Epidemiology, Hokkaido University Research Center for Zoonosis Control, Sapporo 001-0020, Japan; y.eto@frontier.hokudai.ac.jp (Y.E.); atakada@czc.hokudai.ac.jp (A.T.); 3 Laboratory of Parasitology, Graduate School of Infectious Diseases, Faculty of Veterinary Medicine, Hokkaido University, Sapporo 060-0818, Japan; ryo.nakao@vetmed.hokudai.ac.jp; 4 Department of Para-Clinical Studies, School of Veterinary Medicine, the University of Zambia, Lusaka 10101, Zambia; ntongo2004@yahoo.co.uk (E.M.); mudenda68@yahoo.com (B.M.H.); katendi.changula@sacids.org (K.C.); 5 Global Virus Network Affiliate Center of Excellence, the University of Zambia, Lusaka 10101, Zambia; asmweene04@yahoo.com; 6 African Center of Excellence for Infectious Diseases of Humans and Animals, the University of Zambia, Lusaka 10101, Zambia; 7 Department of National Parks and Wildlife, Ministry of Tourism and Arts of Zambia, Lusaka 10101, Zambia; mwizabidaniel@gmail.com; 8 Division of Molecular Pathobiology, Hokkaido University Research Center for Zoonosis Control, Sapporo 001-0020, Japan; h-sawa@czc.hokudai.ac.jp; 9 Department of Disease Control, School of Veterinary Medicine, the University of Zambia, Lusaka 10101, Zambia; martin.simuunza@unza.zm; 10 Global Institution for Collaborative Research and Education (GI-CoRE), Hokkaido University, Sapporo 001-0020, Japan; 11 Global Virus Network, Baltimore, MD 21201, USA; 12 Division of Infection and Immunity, Hokkaido University Research Center for Zoonosis Control, Sapporo 001-0020, Japan; 13 Division of Collaboration and Education, Hokkaido University Research Center for Zoonosis Control, Sapporo 001-0020, Japan

**Keywords:** *Bartonella*, bat fly, bat, PCR, isolation, Zambia

## Abstract

Bat-associated bartonellae, including *Bartonella mayotimonensis* and *Candidatus* Bartonella rousetti, were recently identified as emerging and potential zoonotic agents, respectively. However, there is no report of bat-associated bartonellae in Zambia. Thus, we aimed to isolate and characterize *Bartonella* spp. from bats and bat flies captured in Zambia by culturing and PCR. Overall, *Bartonella* spp. were isolated from six out of 36 bats (16.7%), while *Bartonella* DNA was detected in nine out of 19 bat flies (47.3%). Subsequent characterization using a sequence of five different genes revealed that three isolates obtained from Egyptian fruit bats (*Rousettus aegyptiacus*) were *Ca*. B. rousetti. The isolates obtained from insectivorous bats (*Macronycteris vittatus*) were divided into two previously unclassified bat-associated bartonellae. A phylogenetic analysis of the six genotypes of *Bartonella gltA* sequences from nine pathogen-positive bat flies revealed that three genotypes belonged to the same clades as bat-associated bartonellae, including *Ca*. B. rousetti. The other three genotypes represented arthropod-associated bartonellae, which have previously been isolated only from ectoparasites. We demonstrated that *Ca*. B. rousetti is maintained between bats (*R. aegyptiacus*) and bat flies in Zambia. Continuous surveillance of *Bartonella* spp. in bats and serological surveys in humans in Africa are warranted to evaluate the public health importance of bat-associated bartonellae.

## 1. Introduction

The members of the genus *Bartonella* are Gram-negative, hemotropic, and primary vector-borne bacteria that colonize mammalian endothelial and red blood cells. To date, the genus *Bartonella* consists of more than 30 species, many of which have been described recently [1]. Various mammalian hosts for *Bartonella* spp. have been reported, such as rodents, carnivores, ruminants, and marine mammals [2]. Various arthropods also play important roles in the maintenance and transmission of *Bartonella* spp. In recent decades, several *Bartonella* spp. have been recognized as human pathogens responsible for various clinical manifestations [3]. For example, *Bartonella quintana* is an etiological agent of trench fever, transmitted by louse [4,5], while *Bartonella henselae*, maintained in cat and cat flea, causes cat scratch disease in humans [4,6]. Furthermore, associations between several poorly characterized *Bartonella* spp. and human diseases, including neuroretinitis, febrile illness, fever, and bacteremia, have been reported [7,8,9,10].

Several lines of evidence indicate that bats harbor diverse groups of *Bartonella* species and genotypes, including zoonotic or potentially zoonotic *Bartonella* [11]. *Candidatus* Bartonella mayotimonensis was first detected in the aortic valve tissue of a patient with endocarditis from the United States of America [12]. Subsequently, *Ca.* B. mayotimoensis was detected in little brown bats (*Myotis lucifugus*) and gray bats (*Myotis grisescens*) in Finland [13]. A *Bartonella* genotype detected in bats in Georgia (Caucasus) showed high similarity with *Bartonella* sequences found in the sera of forest workers in Poland [14]. Furthermore, a recent serological study reported the detection of antibodies against *Candidatus* Bartonella rousetti, which was previously detected in bats in Kenya and Nigeria [15,16], among the people who entered the caves to capture bats during a traditional bat festival in southwestern Nigeria [17]. Collectively, these findings strengthen the importance of public health-related studies on bat-associated bartonellae. However, studies about the potential risk of bat-associated bartonellae have not been carried out in many of resource-limited countries. 

The present study intended to characterize *Bartonella* spp. in bats captured in Zambia, where reports on bartonellae are not available. Our results confirmed the presence of several groups of *Bartonella*, both in bats and bat flies in Zambia.

## 2. Results

### 2.1. Isolation of Bartonella from Bats 

**Figure 1:**
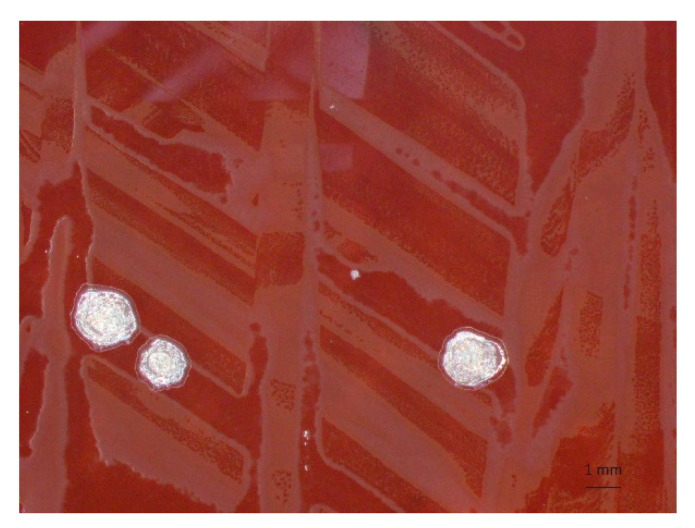
Colonies of *Bartonella* sp. on a blood agar plate. *Bartonella* sp. was isolated from *Rousettus aegyptiacus* (ZB17-79) and cultured on a blood agar plate.

Out of 36 bat blood samples spread on sheep blood agar plates, six samples yielded tiny bacterial colonies with a rough texture and whitish hue during 2 weeks of incubation (Figure 1). Three isolates each were derived from frugivorous bats (*Rousettus aegyptiacus*) (IDs: ZB17-74, -79, and -86) and insectivorous bats (*Macronycteris vittatus*) (IDs: ZB17-107, -109, and -113). All the isolates were confirmed to be *Bartonella* spp. using 16S ribosomal DNA sequencing.

### 2.2. Molecular Characterization of Bartonella Isolates 

Bartonella isolates were characterized via the sequencing of five housekeeping genes encoding citrate synthase (*gltA*), RNA polymerase beta subunit (*rpoB*), transfer messenger RNA (*ssrA*), cell division protein (*ftsZ*), and nicotinamide adenine dinucleotide dehydrogenase gamma subunit (*nuoG*). The sequences of the three isolates obtained from *Rousettus aegyptiacus* (ZB17-74, -79, and -86) were identical for all genes except for *gltA*, in which the isolate ZB17-74 harbored eight nucleotide mismatches. The sequences of all five genes showed the highest sequence identity with those of *Candidatus* Bartonella rousetti deposited in the database (Table 1). The sequences of five genes of two isolates (ZB17-107 and -109) obtained from *Macronycteris vittatus* were identical, which were distinct from those of the other isolate (ZB17-113). These three isolates showed the highest sequence identity with unclassified Bartonella spp. reported from various bat species, namely *Rhinolophus ferrumequinum* from Georgia (*Bartonella* sp. 44552) [14], *Rh. ferrumequinum* from China (*Bartonella* sp. SD-3/2015) [18], *Eidolon helvum* from Kenya (Bartonella sp. B23983) [19], and *Macronycteris commersoni* from Kenya (*Bartonella* sp. H-556) [15], and from a vole (*Clethrionomys rutilus*) from the United States of America (*Bartonella* sp. Cr28649) [20]. 

**Figure 2:**
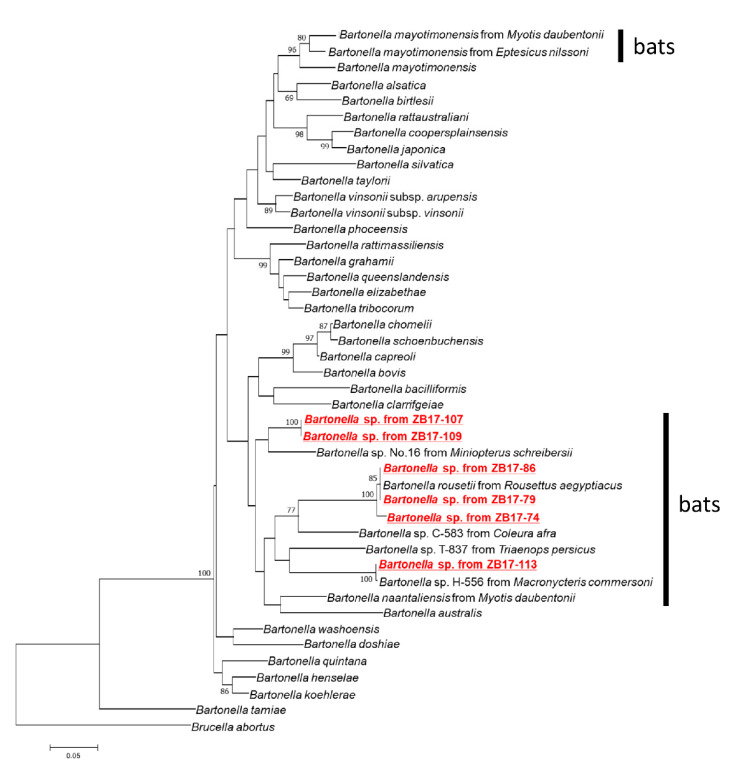
Phylogenetic inference of *Bartonella* spp. A phylogenetic inference from the concatenated sequences of six loci (*ftsZ*, *gltA*, *nuoG*, *rpoB*, *ssrA*, and 16S rDNA) of *Bartonella* species is shown. For phylogenetic reconstruction, the maximum likelihood model proposed in MEGA 6.06 was used with 1000 bootstrap iterations.

A maximum likelihood tree was reconstructed based on the concatenated sequences of *ftsZ*, *gltA*, *nuoG*, *rpoB*, *ssrA*, and the 16S rDNA. In the tree, *Bartonella* detected in this study belonged to a clade, including other *Bartonella* strains detected in bats (Figure 2). Briefly, the isolates from ZB17-74, -79, and -86 formed a cluster with *Candidatus* Bartonella rousetti, while the isolate from ZB17-113 clustered together with *Bartonella* sp. H-556 from *Macronycteris commersoni* from Kenya [15]. The isolates from ZB17-107 and -109 did not cluster with previously reported Bartonella spp., but were phylogenetically closest to Bartonella sp. no. 16 isolated from the common bent-wing bat, *Miniopterus schreibersii*, from Taiwan [21]. 

### 2.3. Detection and Characterization of Bartonella spp. from Bat Flies 

**Figure 3:**
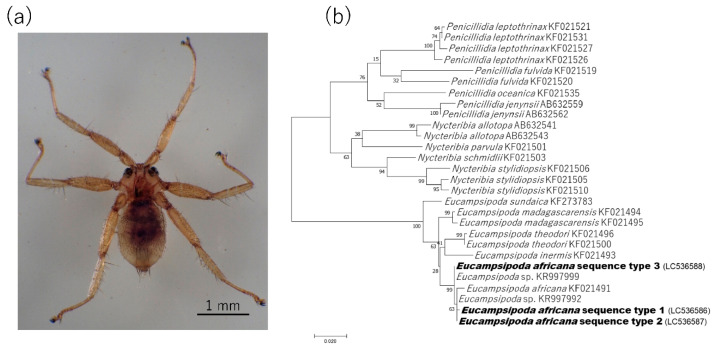
Bat fly and its molecular identification. (**a**) Photograph of a bat fly under an optical microscope. (**b**) A phylogenetic tree based on the partial sequence of a fragment of cytochrome oxidase subunit I (*COI*).

All 19 bat flies were morphologically identified as *Eucampsipoda africana*, which is a common ectoparasite of the Egyptian fruit bat (*Rousettus aegyptiacus*) (Figure 3a). Further analysis of their cytochrome oxidase subunit I (*COI*) sequences divided them into three genotypes (*COI* sequence types 1, 2, and 3), all of which clustered together with *Eu. africana* collected in Kenya (KF021491) (Figure 3b) [22]. In total, nine bat flies were positive for *gltA* in a polymerase chain reaction (PCR). Sequence analysis of the amplicons revealed that the *gltA* sequences from bat flies were of six types. Sequence types 1 and 2 showed 100% (332/332 bp) identity to Bartonella sp. clone NG13-057 isolated from *Eu. africana* of Nigeria (MH151070) and *Bartonella* sp. from *Eucampsipoda* sp. of South Africa (KR997986), respectively [17,23]. Sequence type 3 showed 95.5% (315/330 bp) similarity to the Bartonella symbiont of Eu. theodori from Madagascar (KT751156) [24]. Sequence type 4 showed 99.7% (337/338 bp) similarity to *Candidatus* Bartonella rousetti (HM363764) [15]. Sequence type 5 showed 94.7% (320/338 bp) similarity to Bartonella sp. SD-3/2015 from *Rhinolophus ferrumequinum* of China (KX655838) [18]. The sequence type 6 showed 100% (338/338 bp) identity to *Bartonella* sp. from *Miniopterus schreibersii* of Georgia (KX300183) [14].

**Figure 4:**
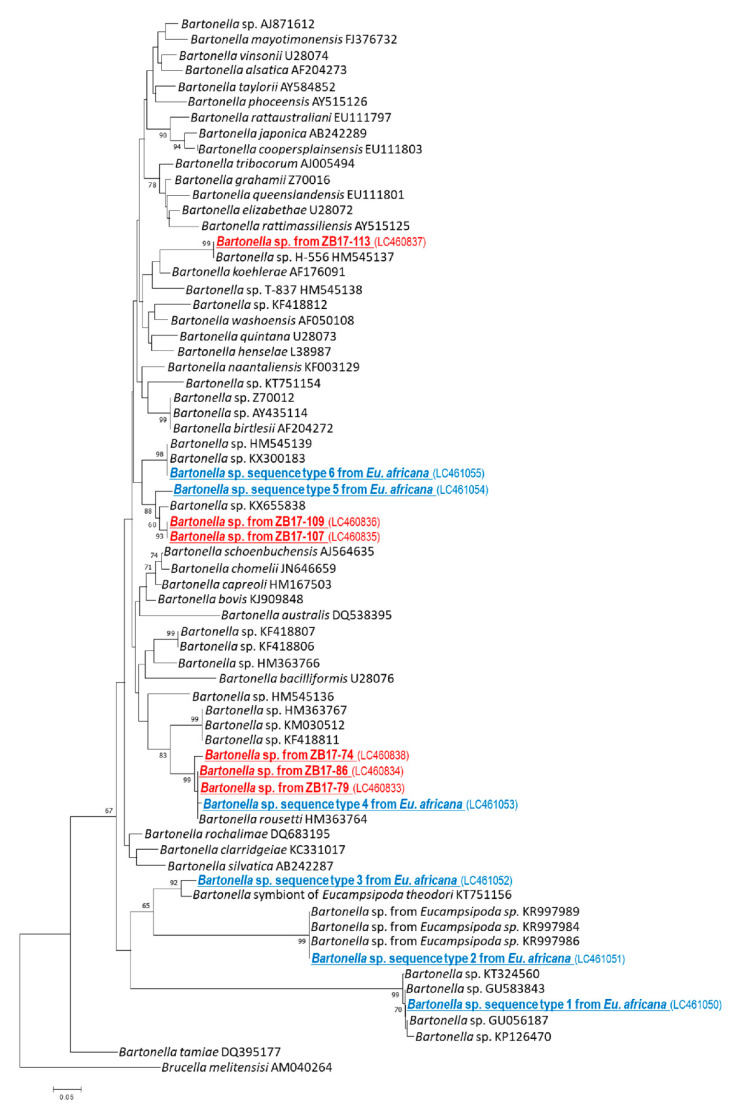
Neighbor-joining phylogeny of citrate synthase (*gltA*) gene. This tree is based on a partial sequence of *gltA* and was rooted with *Brucella melitensis*. Bootstrap values > 60% based on 1000 replications are shown on the interior branch nodes. The sequences from bats and bat flies obtained in this study are shown in red and blue, respectively.

In the phylogenetic tree based on the partial sequences of *gltA*, sequence type 1 clustered together with *Bartonella* spp. from ectoparasites, such as flea and louse in Peru, Tunisia, and Thailand [25,26] (Figure 4). Sequence type 2 clustered together with *Bartonella* spp. isolated from *Eucampsipoda* sp. from South Africa [23]. Sequence type 3 clustered together with symbiotic Bartonella sp. from *Eucampsipoda theodori* of Madagascar [25]. Sequence type 4 was located in the same clade with the isolates from ZB17-74, -79, and -86 and *Candidatus* Bartonella rousetti, which was detected in *Rousettus aegyptiacus* from Kenya [15]. Sequence type 5 clustered together with the isolates from ZB17-107 and -109, and *Bartonella* sp. from *Rhinolophus ferrumequinum* of China. Sequence type 6 clustered together with *Bartonella* sp. from *Miniopterus* sp. of Kenya and Bartonella sp. from Miniopterus schreibersii of Georgia [14,15].

## 3. Discussion

This study investigated the presence and genetic diversity of *Bartonella* in bats and bat flies captured in Zambia in the Southern African region. To the best of our knowledge, this study is the first report on bat-associated bartonellae in Zambia, and the first to isolate *Candidatus* Bartonella rousetti from the Southern African region.

*Candidatus* Bartonella rousetti was first reported as a previously unclassified *Bartonella* strain detected from *Rousettus aegyptiacus* in Kenya in 2010 [15]. Thereafter, the same strain was detected during a survey on *Bartonella* species in bats and bat flies in a cave in southwestern Nigeria, where people entered and captured bats during a traditional bat festival [17]. The study also assessed human exposure to the bacterium using an indirect immunofluorescence assay and detected immunoglobulin G (IgG) against *Ca*. B. rousetti in the sera of several individuals from the surrounding communities without any cross-reaction to other *Bartonella* genotypes [17]. In the present study, *Ca*. B. rousetti was isolated from frugivorous bats (*R. aegyptiacus*) and detected from bat flies (*Eucampsipoda africana*) using PCR. These results indicated that *Ca*. B. rousetti is distributed widely in the African continent and is maintained between bats (*R. aegyptiacus*) and bat flies (*Eu. aficana*) in a cave in Zambia. Local people frequently enter the cave and collect bat feces to use it as manure for their vegetable farms, increasing the risk of *Ca*. B. rousetti infection in the local population, as is the case in Nigeria. Considering that individuals who entered the same cave developed a febrile illness caused by a novel *Borrelia* sp. [27], they should also be tested for bartonellosis for the differential diagnosis of febrile patients living in the vicinity of the cave.

In the present investigation, three isolates were obtained from insectivorous bats (*Macronycteris vittatus*). One isolate obtained from ZB17-113 showed high sequence identities with *Bartonella* sp. H-556 obtained from *Macronycteris commersoni* of Kenya (Table 1). These two isolates clustered together in a phylogenetic tree (Figure 2), suggesting that the same or closely related *Bartonella* species/genotype was infectious to both *M. vittatus* and *M. commersoni*. On the other hand, the sequences of isolates from ZB17-107 and -109 were identical to each other and were distinct from previously reported *Bartonella* spp. These results indicated that at least two distinct *Bartonella* species were prevalent in *M. vittatus* in Zambia. In the previous study, straw-colored fruit bats (*Eidolon helvum*) and long-fingered bats (*Miniopterus* spp.) were found to be positive for three and four different *Bartonella* genotypes, respectively [15]. It is also evident that the same *Bartonella* genotype can be detected from several different bat species [16]. Further investigations on the diversity of bat-associated bartonellae are required to understand the relationship between *Bartonella* spp. and their host bats. Our results provide information regarding the genetic diversity and geographic distribution of bat-associated bartonellae in the African continent.

Bat flies are specially adapted for a nearly permanent ectoparasitic relationship with their host bats. In the present study, we detected six different sequence types of the *Bartonella gltA* from one bat fly species (*Eucampsipoda africana*). A phylogenetic analysis revealed that the sequence types 4, 5, and 6 belonged to the same clades as bat-associated bartonellae, including *Candidatus* Bartonella rousetti (Figure 3). In contrast, the sequence types 1, 2, and 3 clustered together with bartonellae, which were only detected from ectoparasites. Similar findings were also obtained from a previous study conducted in Ghana and in the islands in the Gulf of Guinea, where bat flies harbored not only bat-associated bartonellae, but also other *Bartonella* spp. which were detected only in bat flies [28]. In another study of insect microbiota, arthropod-associated *Bartonella* spp. was reported elsewhere [29,30,31,32]. For example, *Bartonella apis* was isolated from the gut of the honeybee (*Apis mellifera*), which might provide honeybees with resistance to diseases [30,31]. Uncultured *Bartonella* spp. were also detected from Ponerine ants (Hymenoptera: Formicidae: Ponerinae) [32]. These arthropod-associated bartonellae were considered a symbiotic bacterium. A recent study regarding the evolutionary origin of the pathogenic bartonellae suggested that the ancestor of pathogenic bartonellae was a symbiotic bacterium of insects, and that the adaptation to blood sucking arthropods facilitated the colonization of bartonellae in the mammalian blood [33]. This hypothesis may explain why bat flies had more diverse groups of *Bartonella* spp. than bats. 

This present study used a random sampling procedure and samples were collected in one location, which are the limitations of this study. Therefore, the study is not enough to describe the whole picture of bat-associated bartonellae in Zambia. However, the present study revealed the presence of diverse groups of *Bartonella* in bats and their ectoparasites in Zambia. Although cases of human bartonellosis caused by bat-associated *Bartonella* have not been reported so far, further studies such as continuous surveillance of *Bartonella* spp. in bats and serological surveys in humans are warranted to evaluate their potential as zoonotic agents in Africa.

## 4. Materials and Methods 

### 4.1. Ethics 

The capturing of bats was approved by the Department of National Parks and Wildlife (DNPW) and the Ministry of Tourism and Arts of the Republic of Zambia (Act No. 14 of 2015). The approval for placing traps and entering the cave was obtained from the village head and residents. 

### 4.2. Isolation of Bartonella spp. 

In 2017 and 2018, bats were captured in the Leopard’s Hill cave (15.44° S, 28.51° E), Chongwe district, Lusaka province, using a harp trap as a part of a surveillance program of filovirus infection in bats in Zambia, as described in a previous study [34]. Whole blood samples were collected from 36 randomly selected bats (31 *Rousettus aegyptiacus*, four *Macronycteris vittatus*, and one *Rhinolophus* sp.) in vacutainer ethylenediaminetetraacetic acid (EDTA) tubes. Individually, 25 µL of uncoagulated whole blood were spread onto sheep blood agar plates. The plates were incubated at 34 °C with 5.0% CO_2_. 

### 4.3. Characterization of Bartonella spp. 

After two weeks of incubation, bacterial colonies morphologically identified as *Bartonella* were selected and used for DNA extraction using the alkali–heat lysis method. Briefly, a colony was resuspended in 17 µL of 50 mM NaOH and the suspension was heated at 99 °C for 10 min. To adjust pH, 3 µL of 1 M Tris-HCl (pH 7.0) was added. The solutions were used as DNA templates for subsequent genetic characterization.

To confirm the identity of bacterial species, we amplified a fragment of 16S rDNA via PCR using the primers fD1 and Rp2 as described previously [35]. PCRs were conducted in a 20-µL reaction mixture containing 10 µL of Go Taq master mix (Promega, Madison, WI, USA), 100 nM of each primer, and 1 µL of template DNA. The PCR conditions were 95 °C for 2 min and 35 cycles of 95 °C for 30 s, 55 °C for 30 s, and 72 °C for 90 s, followed by a final extension at 72 °C for 5 min. 

For further molecular characterization, PCRs targeting *gltA*, *nuoG*, *ftsZ*, *ssrA*, and *rpoB* were performed as described previously [36,37,38,39,40]. Information regarding the primers used in this study is listed in Table 2. All PCRs were conducted in a 20 µL reaction mixture as described above. The PCR conditions were as described above, with the exception of the annealing temperature (Table 2). The PCR products were visualized after electrophoresis on a 1.2% agarose gel stained with ethidium bromide.

### 4.4. Species Identification of Bat Flies and Detection of Bartonella spp. 

To identify vector arthropods, 19 bat flies were collected from the surface of bat guano in the cave in 2018. The bat flies were morphologically identified to the genus level according to the keys described previously [41], and species were identified by sequencing a DNA fragment of *COI* [42]. DNA was extracted from bat flies using DNAzol (Molecular Research Center, Cincinnati, OH, USA) according to the manufacturer’s instructions. PCR amplification of *gltA* was used to screen *Bartonella*. PCR was performed and the products were analyzed as described above.

### 4.5. Sequencing and Phylogenetic Analysis 

All amplicons were sequenced using the BigDye Terminator version 3.1 cycle sequencing kit (Applied Biosystems, Foster City, CA, USA) and an ABI Prism 3130x genetic analyzer (Applied Biosystems) according to the manufacturer’s instructions. The sequences were analyzed using GENETYX version 9.1 (GENETYX Corporation, Tokyo, Japan) and trimmed on both the 5’ and 3’ ends. The resulting sequences were compared to those in public databases using BLASTn (http://blast.ncbi.nlm.nih.gov/Blast.cgi). A phylogenetic analysis was conducted using MEGA version 6.05 [43]. ClustalW was used to align the sequences to those of closely related organisms deposited in the database. The sequences of multiple loci were concatenated and used for phylogenetic inference of *Bartonella* spp. isolated from bats using the maximum likelihood method. The neighbor-joining method was used to construct a phylogenetic tree based on the *gltA* sequence for the analysis of *Bartonella* spp. from bat flies and bats. The reference sequences used in the phylogenetic inference are listed in Supplementary Table S1. The DNA sequences obtained in this study are available in the GenBank database (accession numbers: LC460827-LC460832 for 16S rDNA, LC460833-LC460838 and LC461050-LC461055 for *gltA*, LC460839-LC460844 for *rpoB*, LC460845-LC460850 for *ssrA*, LC460851-LC460856 for *ftsZ*, LC460857-LC4608622 for *nuoG*, and LC536586- LC536588 for *COI* of *Eucampsipoda africana*).

## Figures and Tables

**Table 1:** Sequence identity of the six isolates to the most closely related *Bartonella* species.

ID	Bat species	*ftsZ*		*gltA*		*unoG*		*rpoB*		*ssrA*	
		Accession Number	BLAST Result	Accession Number	BLAST Result	Accession Number	BLAST Result	Accession Number	BLAST Result	Accession Number	BLAST Result
ZB17-74	*Rousettus aegyptiacus*	HM363769	*Bartonella rousetti* 99%	MH069695	*Bartonella rousetti* B32137 100%	KM387321	*Bartonella rousetti* 99%	HM363774	*Bartonella rousetti* 96%	KM382247	*Bartonella rousetti* 99%
ZB17-79	*Rousettus aegyptiacus*	HM363769	*Bartonella rousetti* 100%	HM363764	*Bartonella rousetti* 100%	KM387321	*Bartonella rousetti* 100%	HM363774	*Bartonella rousetti* 100%	KM382247	*Bartonella rousetti* 100%
ZB17-86	*Rousettus aegyptiacus*	HM363769	*Bartonella rousetti* 100%	HM363764	*Bartonella rousetti* 100%	KM387321	*Bartonella rousetti* 100%	HM363774	*Bartonella rousetti* 100%	KM382247	*Bartonella rousetti* 100%
ZB17-107	*Macronycteris vittatus*	KX300182	*Bartonella* sp. isolate 44552 96%	KX655838	Uncultured *Bartonella* sp. SD-3/2015 98%	KX300163	*Bartonella* sp. isolate 44552 97%	KX300164	*Bartonella* sp. isolate 44552 96%	KM233461	*Bartonella* sp. B23983 97%
ZB17-109	*Macronycteris vittatus*	KX300182	*Bartonella* sp. isolate 44552 96%	KX655838	Uncultured *Bartonella* sp. SD-3/2015 98%	KX300163	*Bartonella* sp. isolate 44552 97%	KX300164	*Bartonella* sp. isolate 44552 95%	KM233461	*Bartonella* sp. B23983 96%
ZB17-113	*Macronycteris vittatus*	KM382254	*Bartonella* sp. H-556 99%	HM545137	*Bartonella* sp. H-556 100%	KM382252	*Bartonella* sp. H-556 100%	EU979536	*Bartonella* sp. Cr28649 88%	KM382250	*Bartonella* sp. H-556 99%

**Table 2:** Primers used in this study.

Primer Name	Sequence 5’-3’	Target Organism	Target Gene	Annealing (°C)	Size Expected (bp)	Reference
fD1	AGAGTTTGATCCTGGCTCAG	*Bartonella* spp.	16S ribosomal DNA	55	1400	35
Rp2	ACGGCTACCTTGTTACGACTT					
BhCS781.p	GGGGACCAGCTCATGGTGG		*gltA*	45	380	36
BhCS1137.n	AATGCAAAAAGAACAGTAAACA					
1400F	CGCATTGGCTTACTTCGTATG		*rpoB*	53	860	40
2300R	GTAGACTGATTAGAACGCTG					
nuoG F	GGCGTGATTGTTCTCGTTA		*nuoG*	55	360	37
nuoG R	CACGACCACGGCTATCAAT					
Bfp1	ATTAATCTGCAYCGGCCAGA		*ftsZ*	55	900	38
Bfp2	ACVGADACACGAATAACACC					
ssrA-F	GCTATGGTAATAAATGGACAATGAAATAA		*ssrA*	60	300	39
ssrA-R	GCTTCTGTTGCCAGGTG					
LCO1490	GGTCAACAAATCATAAAGATATTGG	Bat fly	*COI*	57	710	42
HCO2198	TAAACTTCAGGGTGACCAAAAAATCA					

## Supplementary Materials

The following are available online at https://www.mdpi.com/2076-0817/9/6/469/s1, Table S1: GenBank accession numbers for *ftsZ*, *gltA*, *nuoG*, *rpoB*, and *ssrA*, and 16S rDNA sequences for reference.
